# Serial Magnetic Resonance Imaging in Hypoplastic Left Heart Syndrome Gives Valuable Insight Into Ventricular and Vascular Adaptation

**DOI:** 10.1016/j.jacc.2012.11.016

**Published:** 2013-02-05

**Authors:** Hannah R. Bellsham-Revell, Shane M. Tibby, Aaron J. Bell, Thomas Witter, John Simpson, Philipp Beerbaum, David Anderson, Conal B. Austin, Gerald F. Greil, Reza Razavi

**Affiliations:** ⁎ Division of Imaging Sciences and Biomedical Engineering, Rayne Institute, King's College London, St. Thomas' Hospital, London, United Kingdom; † Department of Pediatric Cardiology, Evelina Children's Hospital, London, United Kingdom; ‡ Department of Pediatric Intensive Care, Evelina Children's Hospital, London, United Kingdom

**Keywords:** hemi-Fontan operation, hypoplastic left heart syndrome, magnetic resonance imaging, Norwood procedure, remodeling of systemic right ventricle, 3D, 3-dimensional, EDV, end-diastolic volume, EF, ejection fraction, ESV, end-systolic volume, HF, hemi-Fontan, HLHS, hypoplastic left heart syndrome, HR, heart rate, iEDV, indexed end-diastolic volume, iESV, indexed end-systolic volume, iSV, indexed stroke volume, LPA, left pulmonary artery, LV, left ventricle, MRI, magnetic resonance imaging, RPA, right pulmonary artery, RV, right ventricle, SENSE, sensitivity encoding, SSFP, steady-state free precession, TCPC, total cavopulmonary connection, TR, tricuspid regurgitation

## Abstract

**Objectives:**

This study sought to investigate changes in magnetic resonance imaging (MRI) ventricular volumes and vascular dimensions before hemi-Fontan (HF) and before total cavopulmonary connection (TCPC) in children with hypoplastic left heart syndrome (HLHS).

**Background:**

The systemic right ventricle (RV) in HLHS is subject to significant changes in volume loading throughout the surgical stages of palliation, particularly after the HF.

**Methods:**

Fifty-eight patients had paired pre-HF and pre-TCPC MRI for assessment of changes of RV volumes, neoaortic flow, and vascular dimensions.

**Results:**

Comparison of pre-HF and pre-TCPC MRI results showed a decrease of indexed RV end-diastolic volume and end-systolic volume (98 ml/m^2^ to 87 ml/m^2^ and 50 ml/m^2^ to 36 ml/m^2^, respectively) with stroke volume remaining constant (49 ml/m^2^ vs. 51 ml/m^2^), leading to an increased RV ejection fraction (51% vs. 59%). These findings persisted after excluding the 3 patients who underwent tricuspid valve repair as part of their HF procedure. Indexed RV end-diastolic volume plotted against neoaortic stroke volume demonstrated a Frank-Starling–like curve that shifted upward after HF. The indexed distal left and right cross-sectional pulmonary artery areas were reduced after HF.

**Conclusions:**

In HLHS, serial MRI shows the adaptation of the systemic RV after HF with volume reduction in the context of a preserved stroke volume and an increased ejection fraction. The staged palliation in HLHS may be a risk factor particularly for reduced left pulmonary artery growth in itself as no factors investigated in this study were found to significantly impact on this.

Hypoplastic left heart syndrome (HLHS) describes the spectrum of left heart under-development, rendering the left side of the heart unable to support the systemic circulation. Before the 1980s, HLHS was universally fatal, with the majority of children dying within 30 days (1). Current management consists of staged palliation. The Norwood procedure is usually performed at a few days of age, combining the aorta and pulmonary artery and creating a systemic to pulmonary artery shunt. Later stages involve connection of the superior vena cava to the pulmonary arteries by hemi-Fontan (HF [with anastomosis to the roof of the right atrium, performed at our institution]) or Glenn (without anastomosis to the roof of the right atrium) followed by routing of inferior vena caval flow to the pulmonary circulation to complete the total cavopulmonary connection (TCPC), leaving the patient with a systemic right ventricle (RV) (2).

Standard investigative management before HF and TCPC has included cardiac catheterization for invasive measurement of pressure and to visualize pulmonary artery anatomy (3), although more recently magnetic resonance imaging (MRI) has been proposed either alone or in conjunction with cardiac catheterization (4,5). Magnetic resonance imaging alone in patients after the Norwood procedure to select candidates to proceed to HF has been suggested (6). A randomized trial did not show any difference between the 2 approaches (7). The use of MRI before HF, and subsequently before TCPC, provides the opportunity to examine the changes in the RV, neoaorta, and pulmonary artery size after the volume reduction after the removal of the systemic to pulmonary artery shunt. An MRI scan can accurately measure ventricular volumes, ejection fraction, and aortic flow. This can be coupled with measurement of the pressure in the internal jugular vein under the same general anesthetic as the MRI scan before TCPC, providing an indication of pulmonary arterial pressure. The proposed MRI approach with central venous pressure measurement in the internal jugular vein may avoid cardiac catheterization in most cases and, therefore, avoid ionizing radiation and the not insignificant morbidities associated with vascular access in these small patients (8,9).

In this study, we investigate the vascular and ventricular changes that occur after HF with serial MRI scans with central venous pressure measurement from the internal jugular vein.

## Methods

### Study population and design

Ethical and institutional approval was obtained (08/H0810/058). Inclusion criteria were all patients with HLHS who had undergone the Norwood procedure, HF, and TCPC from February 2003 to July 2010, and had pre-operative evaluation with cardiac MRI. Patients were identified using the departmental database (Heartsuite XP 3.9.14, Systeria, Glasgow, United Kingdom). The HLHS was defined as any combination of mitral stenosis or atresia with aortic stenosis or atresia (with atrioventricular and ventriculoarterial concordance) necessitating Norwood palliation (2). Patients with unbalanced atrioventricular septal defects or large ventricular septal defects (with balanced ventricles) were excluded. Patient demographics, surgical findings and procedures, and intermediate-term outcome data were collected from the patient case notes and the departmental database and compared to the published literature (4,10–17).

Central venous pressure measurement was performed in all patients pre-TCPC during the same general anesthetic as the MRI. If the central venous pressure was >16 mm Hg, MRI and cardiac catheterization in a hybrid MRI catheter suite was performed, allowing calculation of pulmonary vascular resistance (18). Additionally, some children were referred primarily for MRI cardiac catheterization by the child's principal cardiologist for vascular and pulmonary vascular resistance assessment, if there was severe tricuspid regurgitation, severe right ventricular dysfunction on echocardiography, previous total anomalous pulmonary venous drainage, or significant airway pathology.

### Image acquisition and analysis

All MRI scans were performed on a Philips 1.5-T Achieva Scanner (Philips Healthcare, Best, the Netherlands) under general anesthetic. The coil was selected according to the patient's size. Either a small or medium 2-element flexible coil (Flex-S or Flex-M, Philips Healthcare) was used in neonates and infants, respectively. In patients weighing >15 kg, a standard 5-element cardiac coil (3 anterior and 2 posterior elements) was used. Four-electrode vector electrocardiography was used for cardiac triggering. All patients were examined supine with vector electrocardiographic leads on the anterior hemithorax. After a survey and sensitivity encoded (SENSE) reference scan, a real-time, interactive, steady-state-free precession (SSFP) sequence was used to identify the imaging planes that would be used in subsequent scans.

All cine images were electrocardiography triggered and performed with cessation of ventilation for the duration of each acquisition. First, for assessment of the cardiac rest period and the subjective degree of tricuspid regurgitation (TR), a 4-chamber view in high temporal resolution was acquired with a retrospective vector electrocardiography-gated SSFP 2-dimensional cine sequence (TR = 3.1 to 3.6 ms, TE = 1.6 to 1.8 ms; acceleration factor [SENSE acquisition] 2, 60° flip angle, 200 to 320 mm field of view [FOV], slice thickness 4 to 6 mm, inplane resolution 1.3 to 2.0 mm; temporal resolution 60 to 80 phases, breath-hold duration 11 to 15 s). Reducing the temporal resolution of this sequence, short-axis cuts for assessment of ventricular function were acquired (10 to 14 slices; 5 to 7 breath-holds, each 11 to 15 s long; 30 to 40 cardiac phases). The extracardiac vasculature was imaged then, using a first-pass 3-dimensional (3D) angiography technique after intravenous injection of an extravascular contrast agent (either gadopentetate dimeglumine [Magnevist, Berlex Laboratories, Wayne, New Jersey] or gadoterate meglumine [Dotarem, Guerbet, Villepinte, France]), 0.1 mmol per kilogram body weight; 200 to 320 mm FOV, 1.2 to 1.7 mm isotropic voxel size, acceleration factor (SENSE acquisition) 2, flip angle 40°, breath-hold time 12 s to 17 s per phase (2 phases acquired).

Subsequently, a 3D SSFP whole-heart MR coronary angiography sequence was applied for imaging of the great vessels and cardiac anatomy. Navigator gating (gating window 3.0 mm, mean navigator efficiency of 55%) was used to compensate for respiratory motion in our anesthetized patients. For contrast enhancement, a flow-insensitive T2-prepulse for suppression of myocardial signal and a spectrally selective fat saturation pulse were used (19,20). Other imaging parameters included a 200- to 320-mm FOV, TR = 4.1 to 5.2 ms, TE = 2.1 to 2.5 ms, 90° flip angle, acceleration factor (SENSE acquisition) 2, and 70 to 110 sagittal slices with an isotropic voxel size of 1.2 to 1.5 mm (19,20). This was followed by a free breathing phase contrast flow magnetic resonance imaging sequences with retrospective electrocardiography gating for the assessment of flow volumes (TR = 4.6 ms, TE = 2.8 ms, 15° flip angle, 3 signal averages, acceleration factor [SENSE acquisition] 2, slice thickness 4 to 6 mm, FOV 200 to 320 mm, 40 cardiac phases, in-plane resolution 1.0 to 1.5 mm, through-plane velocity encoding values 50 cm/s to 100 cm/s [in the venous hemi-Fontan or TCPC pathways] or 150 to 200 cm/s [in the systemic arterial vessels]).

#### Volumetry

The RV end-diastolic volume (EDV) and end-systolic volume (ESV), stroke volume (SV), and ejection fraction (EF) were calculated from a stack of short-axis 2-dimensional SSFP cine images using the disc summation method after tracing endocardial contours in end diastole and end systole.

#### Flow Measurements

Phase contrast flow measurements were analyzed to calculate neoaortic, superior caval vein, and in pre-TCPC patients, branch pulmonary artery flow. Superior caval vein flow was obtained below the innominate and measured bilaterally if the patient had bilateral superior caval veins. Neoaortic flow was measured below the level of the Damus-Kaye-Stansel anastomosis. Differential pulmonary blood flow was calculated from independent branch flows or in some patients with only superior caval vein and 1 branch pulmonary artery flow from right pulmonary artery (RPA) = superior caval vein minus left pulmonary artery (LPA) or LPA = superior caval vein minus RPA. All volumes and flows were indexed to body surface area (21). Differential branch pulmonary flows were not done before HF as turbulent and fast flow patterns from an aortopulmonary shunt in small pulmonary vessels make it very difficult to obtain reliable flow results.

#### Vessel Measurements

**Figure 1:**
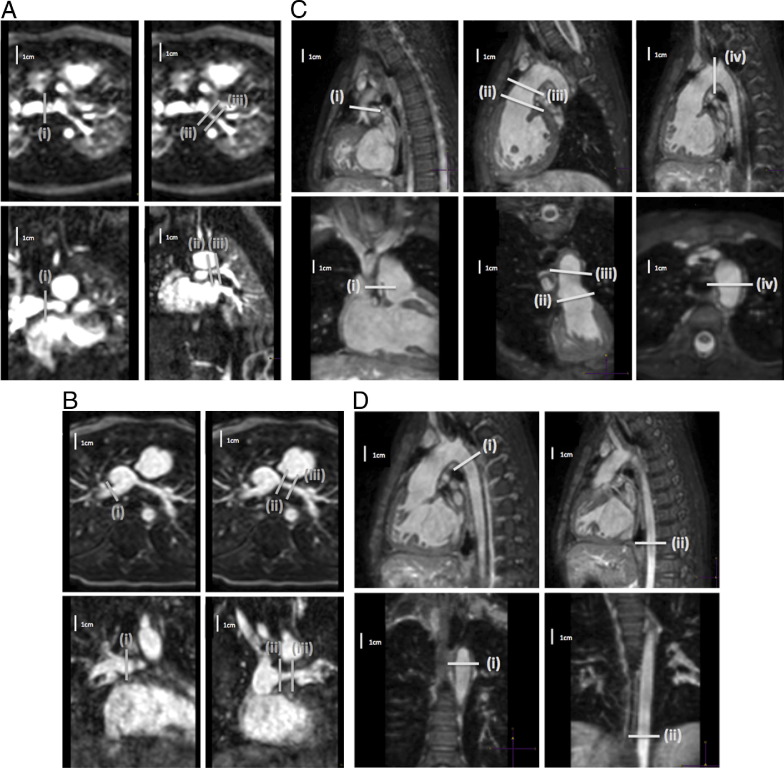
Measurement of Branch Pulmonary Arteries After Norwood and Hemi-Fontan, and Assessment of Proximal and Distal Aortic Dimensions **(A)** Measurement of branch pulmonary arteries after the Norwood procedure showing the 2 planes for measuring (i) distal right pulmonary artery; (ii) proximal left pulmonary artery; and (iii) narrowest left pulmonary artery. **(B)** Measurement of branch pulmonary arteries after the hemi-Fontan showing the 2 planes for measuring (i) distal right pulmonary artery; (ii) proximal left pulmonary artery; and (iii) narrowest left pulmonary artery. **(C)** Assessment of proximal aortic dimensions showing the 2 planes for measuring (i) native aorta; (ii) neoaortic root; (iii) ascending aorta; and (iv) transverse arch. **(D)** Assessment of distal aortic dimensions showing the 2 planes for measuring (i) upper descending aorta; and (ii) descending aorta at the diaphragm.

Gadolinium-enhanced angiography was preferred for the branch pulmonary artery measurements as the authors found this provided better image quality for small branch pulmonary arteries than 3D SSFP. For larger vessels, both techniques have been shown previously to yield similar results (22). Branch pulmonary artery and aortic areas were measured (Fig. 1). Areas were assessed using multiplanar reformat and indexed to body surface area (23). The ratio of the narrowest measurement in the LPA and the proximal LPA was calculated, as well as the ratio of the upper descending aorta and the diaphragmatic aorta.

#### Evaluation of Residual LV Morphology

As the effect on the RV was more likely to be due to the residual morphology of the left ventricle (LV) than the presence/absence of forward flow, LV morphology was described as follows: no LV visible or slitlike LV; globular LV; or borderline LV. Borderline LV was defined as a more mildly hypoplastic LV that could be considered potentially able to support the systemic circulation (2). In general, the no visible LV or slitlike LV corresponded to the mitral atresia and aortic atresia subgroup, the globular LV corresponded to the mitral stenosis and aortic atresia subgroup, and the borderline LV to the mitral stenosis and aortic stenosis subgroup.

#### Tricuspid Regurgitation

Tricuspid regurgitation was qualitatively classified as none/trivial/mild or moderate/severe from the 2-dimensional SSFP cine images. If quantitative data were available, tricuspid regurgitant fraction was calculated from ventricular stroke volume (from short-axis stack) less neoaortic forward flow (from phase contrast flow) and expressed as a percentage of the ventricular stroke volume. Patients with large ventricular septal defects were excluded from the quantitative assessment of TR because of the unquantified shunt across the ventricular septal defect.

#### Collateral Vessels

The presence and type of collateral vessels (aortopulmonary, venovenous, or arteriovenous) were recorded on the basis of a review of gadolinium-enhanced angiography and 3D SSFP images assessed using multiplanar reformat. This is a retrospective study, and aortopulmonary collateral flow was not quantified. All analyses (flows and volumes) were performed using Viewforum EWS version 2.0 (Philips Healthcare, Best, the Netherlands) by 2 members of the research team (H.B.R., A.B.), blinded to each other's analysis. Observers were blinded to the clinical data.

#### Central Venous Pressure Measurement

Central venous pressure was measured using a water manometer connected to a cannula in the internal jugular vein. The measurements were converted from centimeters of water to millimeters of mercury. Patients who underwent MRI cardiac catheterization had direct pressure measurements of the branch pulmonary arteries with wedge pressures to calculate the transpulmonary gradient and, combined with MRI phase contrast flow, pulmonary vascular resistance (18) using the equation: pulmonary vascular resistance = (mean pulmonary artery pressure minus pulmonary wedge pressure) divided by (pulmonary blood flow divided by body surface area). Interventions under MRI guidance were not performed in this study group.

### Statistics

Statistical analyses were undertaken using Stata version 11 (StataCorp, College Station, Texas). Bivariate comparisons comprised paired *t* tests and Wilcoxon signed-rank tests as appropriate. Interuser variability of the right ventricular volume measurements was quantified using an intraclass correlation coefficient 2-way model with absolute agreement (also known as ICC[2,1]), whereby each single measurement was assessed by 2 authors (H.B.R., A.B.) (24). Linear regression modeling was used to quantify factors associated with change in volumetric parameters between operative stages. Variables were chosen based on clinical considerations, and all were tested simultaneously (i.e., full models only were used). Partial r^2^ values were reported to quantify the contribution of individual predictors to overall model fit. Model stability was checked using residual versus fit and leverage versus residual squared plots. Outliers were screened by calculation of Cook's distance and standardized dfit betas. Functional form of predictors was assessed using augmented component-plus-residual plots. A p value of <0.157 was classified as significant within the regression models (this value maximizes model prediction, as it is based upon penalty criteria, such as Akaike Information Criteria and Mallows Cp).

Of note, all model stability and outlier checks outlined above revealed no problems.

## Results

**Figure 2:**
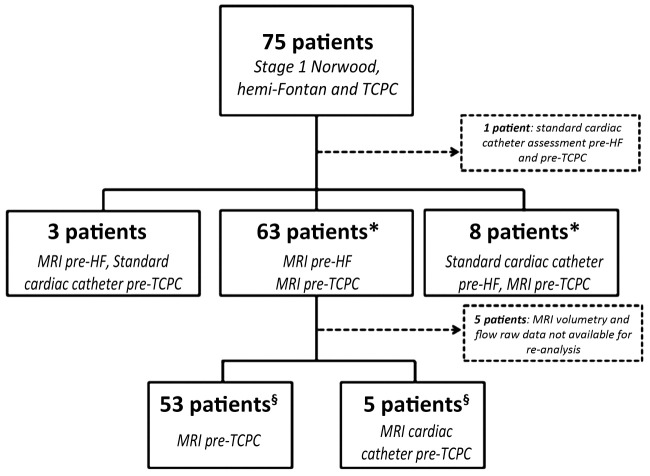
Patients Undergoing TCPC Between February 2003 and July 2010 at Evelina Children's Hospital The 71 patients indicated by (*) had magnetic resonance imaging (MRI) before total cavopulmonary connection (TCPC). The 58 patients indicated by **(§)** were used to assess remodeling before and after hemi-Fontan (HF). Five patients had assessment of pulmonary vascular resistance in a combined MRI-cardiac catheterization procedure.

Between February 2003 and July 2010, 75 patients with HLHS have undergone the Norwood procedure, HF, and TCPC at our institution (Fig. 2). Three patients were assessed with MRI before HF and standard cardiac catheter before TCPC, and 1 was assessed before HF and TCPC by standard cardiac catheter. Therefore, 71 patients underwent assessment with cardiac MRI before TCPC. Of the 71 patients assessed before TCPC with MRI (n = 66) or MRI cardiac catheterization (n = 5), 63 patients had cardiac MRI assessment before HF additionally. Five patients did not have volumetry and flow raw data available for reanalysis. The serial data of the 58 remaining patients were used for assessing remodeling before and after HF, and are described in this paper. All patients assessed in this era were deemed suitable for TCPC.

Demographics and cardiovascular morphology for the patients are described in Tables 1 and 2. Interstage procedures are recorded in Table 3. All patients had undergone the classical Norwood procedure with creation of an aortopulmonary Damus-Kaye-Stansel anastomosis, reconstruction of the aortic arch, and an insertion of a 3.5 or 4.0 mm modified Blalock-Taussig shunt between the innominate artery and RPA. After that, a HF and fenestrated lateral tunnel TCPC was performed.

### Interobserver variability of MRI analysis

There was good agreement between the 2 observers. Intraclass coefficient (95th confidence interval) was 0.945 (0.741 to 0.91), 0.952 (0.779 to 0.984), 0.926 (0.780 to 0.970), 0.885 (0.764 to 0.946), and 0.999 (0.997 to 0.999) for EDV, ESV, SV, EF, and neoaortic stroke volume, respectively.

### Stroke volume validation

Bland-Altman comparisons for the stroke volumes derived by the 2 methods (volumetric assessment of the ventricle and phase contrast flow imaging in the neoaorta) agreed closely. Before HF, the mean bias and precision (expressed as a percentage of the averaged stroke volume for each patient) was 1.9 ± 11.9%; before TCPC, the figure was 3.8 ± 8.5%.

### Ventricular remodeling

The volume reduction after HF resulted in a significant fall in RV cardiac index (5.5 l/min/m^2^ to 4.5 l/min/m^2^, an 18% fall) (Table 4). Indexed RV stroke volume (iSV) remained relatively constant, but there was a significant fall in heart rate (HR). There was also a fall in RV indexed end diastolic volume (iEDV) and indexed end systolic volume (iESV [11% and 27%, respectively]), resulting in an overall increase in the mean RV EF from 51% to 59%.

A linear regression model (Table 5) was used to look at the factors that contribute to changes in the iEDV and iESV before and after HF. The factor with the biggest effect on change in iEDV (or iESV) was the pre-HF iEDV (or iESV), accounting for two-thirds of the variability within the model (partial R^2^ 0.68), with ventricular morphology and degree of TR (assessed subjectively on cine images) pre-HF contributing to the bulk of the remainder. After adjustment for the other variables, the pre-HF indexed volumes contributed on average to a 12% drop in iEDV (coefficient −0.71) and a 24% drop in iESV (coefficient −0.70), meaning that the largest absolute fall was seen in patients with the most dilated ventricles.

Patients with a borderline LV that is still contributing to cardiac output also appeared to have a greater degree of remodeling, with on average an additional 20 ml fall in iEDV after HF. This was seen to a lesser degree in those with a globular LV morphology. There was no significant difference in residual LV volumetry after hemi-Fontan in patients with a borderline LV.

Presence of aortopulmonary collaterals pre-HF had a negative impact on remodeling, on average adding 6.2 ml to the iEDV. Age at HF was not associated with a change in iEDV or iESV, nor was the use of angiotensin-converting enzyme inhibitor therapy at the time of the pre-HF scan.

**Figure 3:**
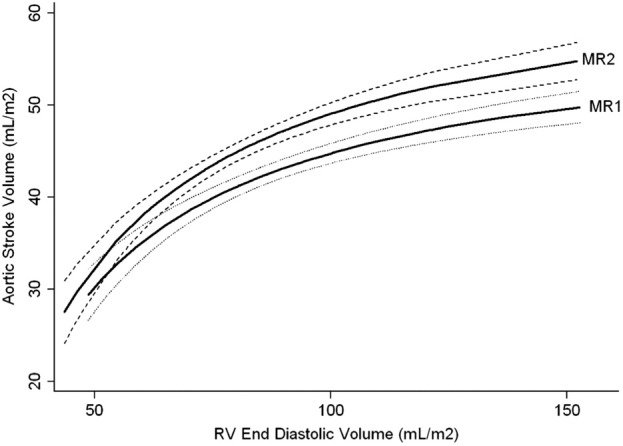
Indexed RV EDV Plotted Against Indexed Neoaortic Stroke Volume Indexed right ventricular (RV) end-diastolic volume (EDV) plotted against indexed neoaortic stroke volume showing an upward and leftward shift in the curve (which resembles the Frank-Starling curve) after hemi-Fontan (HF), suggesting improved efficiency. Curves are calculated using fractional polynomial regression based on the 58 paired magnetic resonance imaging (MRI) scans; **solid lines** = predicted fit, **dashed lines** = 95% confidence intervals. MRI1 is pre-HF; MRI2 is pre-total cavopulmonary connection.

**Figure 4:**
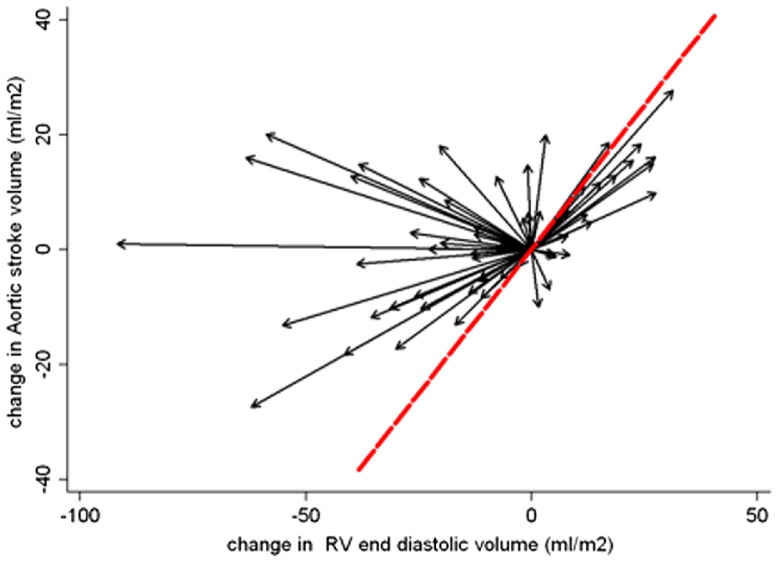
Within-Patient Change in Loading Conditions, RV iEDV, and iSV Between MRI1 and MRI2 Change in loading conditions, right ventricle (RV) indexed end-diastolic volume (iEDV), and indexed stroke volume (iSV) within individual patients (n = 58) between magnetic resonance imaging (MRI) scan 1 (MRI1), before hemi-Fontan (HF), and MRI2, before total cavopulmonary connection (TCPC). Each patient acts as his or her own control with the baseline volumes for each patient before HF zeroed to the reference point (0,0). Thus, each vector relates to the change in volume loading for an individual patient between MRI1 and MRI2, with an improvement in cardiac efficiency highlighted by vectors traveling to the left of the line of identity, which occurs in the majority of patients (67%).

The group-averaged relationship between iEDV and indexed neoaortic stroke volume before and after HF (n = 58) is described in Figure 3. The regression line of best fit for the group as a whole can be interpreted as consistent with the Frank-Starling mechanism (25), which demonstrates a shift to the left and upward after the HF (i.e., MR1 vs. MR2). This is also shown in Figure 4, whereby changes in iEDV versus stroke volume are shown for individual patients (i.e., each patient acts as his or her own control). An improvement in cardiac efficiency is highlighted by vectors traveling to the left of the line of identity, which occurs in the majority of patients (67%). This is consistent with the improvement in ejection fraction highlighted in Table 4.

### Tricuspid regurgitation

To improve statistical power TR was assessed subjectively on 2-dimensional SSFP cine images in the 58 paired MRI examinations as not all MRI examinations provided enough quantitative data for TR calculation. The presence of moderate or severe TR pre-HF contributed on average an increase of 16 ml to the iEDV. Qualitative assessment of TR calculation correlated with quantitative assessment, which was available in 9 patients with moderate (20% to 40%) or severe (>40%) TR (regurgitant fraction 27% to 72%, mean 38%). Three of these patients (regurgitation fractions 37%, 68%, and 24%, respectively) underwent tricuspid valve repair at HF. To address the potential influence of these 3 patients on ventricular remodeling, we performed a sensitivity analysis by rerunning the regression model seen in Table 5. After exclusion of these 3 patients, the model fit did not change significantly (r^2^ went from 0.79 to 0.77), and neither the significance levels nor the partial r^2^ values for individual variables changed appreciably. Similarly, the relative changes in coefficients were modest, ranging from −10% to +25%. Thus, we can conclude that the influence of tricuspid valve repair did not have a significant influence on ventricular remodeling. However, this finding must be interpreted with caution, because of the small number of patients who underwent tricuspid valve repair (n = 3).

### Vascular remodeling

Pulmonary artery and aortic dimensions are summarized in Table 6 and the Online Table 1. Measurement of the RPA cross section is challenging after the HF due to the proximity of the anastomosis to the RPA branching, and so measurements of the distal RPA were not possible in all cases (Table 6). The indexed LPA area increased proximally but reduced distally. The indexed RPA area also reduced after the HF. Mean differential flow pre-TCPC was 63.0% to the right and 37.0% to the left (Table 4). There was a significant difference in LPA flow, with reduced flow in those with the ratio of the narrowest section of the LPA to the proximal LPA <50%. There was no correlation between the narrowest area of the LPA and superior vena cava iSV; however, superior vena cava iSV correlated positively with distal RPA size (Pearson 0.550, p = 0.004).

We examined whether LPA size at the narrowest point (after adjustment for age) at MR1, MR2, or change in LPA size (after adjustment for age and presence of left pulmonary arterioplasty before MR2) was associated with a range of anatomical and flow-related factors. Anatomical factors included ventricular dilation (iEDV) and proximal aortic area (neoaorta and native aorta). Flow-related factors included total cardiac output (pre-HF only), presence of shunt narrowing (pre-HF only), and collaterals (both aortopulmonary and venovenous). Left pulmonary artery growth was weakly associated positively with proximal aortic area growth; however, no other factor was consistently associated with LPA narrowing.

There was no significant difference in the indexed sizes of the native aorta, transverse aortic arch, and proximal descending aorta after HF, even once patients with residual coarctation requiring repair between the pre-HF and pre-TCPC MRI scans are accounted for. The indexed area of the descending aorta at the level of the diaphragm, however, significantly reduces after the HF. However, that was neither correlated with reduced cardiac output (r = 0.10, p = 0.45) nor with reduced stroke volume (r = 0.10, p = 0.46).

### Central venous pressure

There was no correlation between central venous pressure and any of the measured ventricular or vascular variables with correlation coefficients, ranging from −0.22 to 0.16 (all p > 0.12).

### Intermediate-term outcomes

Median follow-up was 766 days (interquartile range 230.5 to 1,319 days). Current life status was checked by using their National Health Service number. There was 1 early mortality (<24 h) from an intractable arrhythmia. No patients required takedown of the TCPC, extracorporeal membrane oxygenation, or transplantation. Three patients have protein-losing enteropathy, and 2 of these patients have required LPA stenting (228 and 320 days after TCPC) to optimize hemodynamics. One patient had a permanent pacemaker implanted for symptomatic bradycardia related to nodal rhythm, and another required cardioversion for 1 episode of atrial flutter.

## Discussion

The patient cohort observed in this study showed that serial MRI in HLHS provides comprehensive information on right ventricular function and vascular morphology.

An interesting observation is the intermediate-term outcome of those patients assessed with MRI coupled with central venous pressure measurement without cardiac catheterization. Our results are comparable to the published literature (4,10–17). Therefore, this approach may be an effective and safe way to assess suitability for TCPC. It provides comprehensive information on ventricular function and vascular morphology, reserving an invasive hemodynamic study for a selected group of high-risk patients, and thereby minimizing exposure to ionizing radiation and the complications of vascular access.

The range of right ventricular volumes was wide, encompassing patients with moderate or severe TR and smaller volumes in those with significant forward flow through the left heart. The reduction in volume loading after HF, with iESV and iEDV both falling as expected, led to an increase in EF, although there were no factors that predicted the increase in EF. A reduced fall in ventricular volumes was associated with significant TR as well as the presence of aortopulmonary collaterals, both of which are known to volume load the ventricle. As the majority of patients (84%) in this group had 3.5-mm shunts, it was not possible to evaluate the effect of shunt size.

It was noted that nearly all of the patients with moderate or severe TR before HF also had moderate or severe TR before TCPC, and so persistent TR would explain this adverse remodeling and increase in iEDV (even after excluding the 3 patients who underwent tricuspid valve repair as part of their HF procedure). This also suggests that the moderate or severe TR is not purely a result of annular dilation, but an inherent abnormality of the tricuspid valve. Interestingly, age at HF was not significantly associated with greater reduction in ventricular volumes and suggests that early surgery does not appear to be indicated solely to help with the post-HF remodeling process.

Normal ranges for MRI EF have previously been described in the post-TCPC single ventricle setting (26), but we have demonstrated the impact of operative stage, which should additionally be taken into account.

There was a marked discrepancy in the relative sizes of the RPA and LPA, with the proximal LPA being approximately two-thirds of the size of the RPA past the HF anastomosis. This discrepancy was more marked before HF. The mechanism of this is likely to be related to the change in the flow dynamics of pulmonary blood flow, for example, from an arterial shunt in the RPA directing more of the flow into this vessel, to the lower pressure HF anastomosis redistributing the blood more evenly. Of note, the superior vena cava iSV positively correlated with the RPA size.

Left pulmonary artery stenosis is known to be a significant problem in HLHS (2). In this group, we found no significant factors affecting LPA growth. However, this may be that LPA narrowing in HLHS after staged palliation is a universal problem, and that the staged palliation is in itself a risk factor. Left pulmonary artery size will need close monitoring in this patient cohort, and some of these patients may still require intervention to address this in the future. The timing of intervention should take into account the potential for future growth of the LPA bed in the setting of significant proximal stenosis.

There was no aortic narrowing in any of the patients pre-TCPC. The 3D MRI images show that the areas reconstructed in the initial Norwood surgery (proximal neoaorta, transverse arch, and proximal descending aorta) did not remodel after HF. That is likely to be related to reduced compliance of these vessels secondary to the incorporated homograft tissue. This extends beyond the point where the duct was attached by at least 5 mm into the proximal descending aorta. Conversely, the proximal descending aorta is already larger after surgery than it might be expected in a normal aorta. At the diaphragm, no operation was performed and, therefore, the aorta is growing at a normal pace.

As there is significant somatic growth between the 2 MRI scans, areas indexed to body surface area were chosen for statistical analysis. Recently, Voges et al. (27) showed reduced aortic wall distensibility in similar areas in 40 older patients who had Norwood surgery for HLHS. In our patients, the aorta at the level of the diaphragm did remodel with a reduction in area/m^2^, which is probably due to the reduced cardiac output through the aorta after HF.

Changes in indexed neoaortic stroke volumes versus end-diastolic volumes across the whole cohort resembled the Frank-Starling relationship (Fig. 3). This curve showed an upward shift comparing the pre-TCPC data with pre-HF (Figs. 3 and 4). The HF may, therefore, confer a benefit in RV contractility across the population. As contractility was not directly measured in this study, the shift in the curve may also be attributed to other causes such as changes in systemic vascular resistance, which was not assessed in our patients.

### Study limitations

Pulmonary to systemic blood flow ratio was not assessed in the pre-HF patients, and aortopulmonary flow was not quantified. Therefore, the relation of these to RV remodeling could not be evaluated quantitatively. The MRI scans were performed under general anesthetic, which can affect systemic and pulmonary vascular resistances, particularly in the single ventricle setting when the patient is supine with positive pressure ventilation. For assessment of outcome using MRI coupled with central venous pressure, no control group using cardiac catheterization only for pre-operative evaluation was available.

## Conclusions

Performing MRI scans before HF and TCPC allows an insight into vascular and right ventricular remodeling of the systemic RV. The HF operation appears to not only volume off-load the ventricle (with significant reduction in RV iEDV and iESV) but may also improve contractility. The staged palliation in HLHS may be a risk factor particularly for reduced LPA growth in itself, as no factors investigated in this study were found to have a significant impact on this.

## Figures and Tables

**Table 1:** Demographics and Morphological Details, n = 58

Male	40 (69%)
Antenatal diagnosis	52 (91%)
Gestation >37/40	54 (96%)
Normal atrial situs	58 (100%)
Apex to the left	58 (100%)
Left ventricular morphology	
No visible/slitlike LV	25 (43%)
Globular LV	27 (47%)
Borderline LV⁎	6 (10%)
Ventricular septal defect	4 (7%)
Bilateral superior caval vein	6 (10%)
Norwood procedure shunt	
3.5 mm	48 (84%)
4.0 mm	9 (16%)
Birth weight, kg	3.06 ± 0.45

Values are n (%) or mean ± SD. Chi-square used for categorical variables, Student *t* test for continuous variables.

⁎ Borderline left ventricle (LV) is a left ventricle that is mildly hypoplastic and may be considered potentially able to support the systemic circulation.

**Table 2:** Mean Age, Weight, and Saturations, n = 58

	Age, yrs (SD)	Weight, kg (SD)	Saturations, % (SD)
Norwood procedure	0.01 (0.02)	3.07 (0.42)	—
Pre-HF MRI	0.44 (0.18)	5.80 (1.27)	76.5 (7.2)
HF	0.58 (0.20)	6.45 (1.47)	—
Pre-TCPC MRI	2.91 (0.81)	13.01 (2.11)	82.1 (5.0)
TCPC	3.45 (0.89)	14.02 (2.13)	—

HF = hemi-Fontan; MRI = magnetic resonance imaging; TCPC = total cavopulmonary connection.

**Table 3:** Interstage and Associated Procedures, n = 58

	Pre-HF	At HF	Pre-TCPC⁎	At TCPC
Aortic arch†	4 (6.9%)	11 (19.0%)	0 (0.0%)	0 (0.0%)
Pulmonary arteries	2 (3.4%)	4 (6.9%)	0 (0.0%)	0 (0.0%)
Tricuspid valve	0 (0.0%)	1 (1.7%)	2 (3.4%)	11 (19.0%)
Collateral occlusion‡	0 (0.0%)	2 (3.4%)	3 (5.2%)	1 (1.7%)
Other§	2 (3.4%)	1 (1.7%)	3 (5.2%)	0 (0.0%)

Values are n (%). All pre-HF procedures were catheter procedures. Abbreviations as in Table 2.

⁎ Pre-TCPC: surgical relief of pulmonary vein stenosis and ligation of collateral; plication of eventration of the right diaphragm.

† The 4 patients who had aortic arch balloons before HF are included in the 11 who had an arch repair at HF.

‡ One patient had venovenous collaterals occluded at HF and then venovenous collaterals and aortopulmonary collaterals occluded before TCPC.

§ Before HF: shunt stent, atrial septal stent; at HF: reimplantation of the innominate artery.

**Table 4:** Volumetric and Flow Data (n = 58)

	Pre-HF MRI		Pre-TCPC MRI		
	Mean ± SD	Range	Mean ± SD	Range	p Value
Heart rate, beats/min⁎	113.5 ± 14.7	83–150	89.3 ± 16.0	57–133	<0.001
Indexed EDV, ml/m^2^⁎	98.1 ± 35.5	48.7–222.6	87.2 ± 22.4	43.7–163.8	0.002
Indexed ESV, ml/m^2^⁎	49.5 ± 24.4	15.3–126.5	36.0 ± 12.9	11.0–72.9	<0.001
Indexed SV, ml/m^2^	48.6 ± 14.7	19.9–105.6	50.9 ± 12.9	32.0–98.7	0.22
Cardiac index, l/min/m^2^⁎	5.5 ± 2.0	2.5–13.4	4.5 ± 1.4	2.8–8.9	<0.001
Ejection fraction, %⁎	51.1 ± 8.8	28.4–72.4	59.3 ± 8.1	43.2–79.3	<0.001
Right SCV SV, ml/beat/m^2^ (n = 53)	—	—	19.8 ± 6.7	6.3–36.8	—
Left SCV SV, ml/beat/m^2^ (n = 4)	—	—	10.2 ± 8.6	2.4–22.0	—
RPA SV, ml/beat/m^2^ (n = 41)	—	—	12.7 ± 4.9	2.5–25.3	—
LPA SV, ml/beat/m^2^ (n = 35)	—	—	7.3 ± 3.2	1.0–13.2	—
RPA flow, % (n = 51)	—	—	63.0 ± 10.7	28.3–89.8	—
LPA flow, % (n = 51)	—	—	37.0 ± 10.7	10.2–71.7	—
CVP, mm Hg (n = 50)	—	—	12.1 ± 2.3	6.0–16.0	—

CVP = central venous pressure; EDV = end-diastolic volume; ESV = end-systolic volume; LPA = left pulmonary artery; RPA = right pulmonary artery; SCV = superior caval vein; SV = stroke volume; other abbreviations as in Table 2.

⁎ Significant, paired Student *t* test in 58 patients.

**Table 5:** Model for Change in Indexed End-Diastolic Volume (r^2^ = 0.79, adjusted r^2^ = 0.75), n = 58

	Coefficient (95% CI)	p Value	Partial r^2^
Pre-HF iEDV	−0.71 (−0.86 to −0.57)	0.000⁎	0.68⁎
Borderline LV morphology	−20.12 (−33.01 to −7.23)	0.003⁎	0.17⁎
Moderate/severe TR on pre-HF MRI	16.19 (4.61 to 27.78)	0.007⁎	0.14⁎
Globular LV morphology	−6.54 (−14.20 to 1.11)	0.09⁎	0.06⁎
Aortopulmonary collaterals seen on pre-HF MRI	6.23 (−0.97 to 13.44)	0.09⁎	0.06⁎
Age at HF	−7.53 (−26.75 to 11.68)	0.43	0.01
Pre-HF EF	−0.19 (−0.73 to 0.34)	0.47	0.01
ACEI therapy at pre-HF MRI	1.83 (−5.65 to 9.30)	0.63	0.01
Time between HF and pre-TCPC MRI, yrs	−0.40 (−4.80 to 4.00)	0.86	—

⁎ Significant results.

**Table 6:** Pulmonary Artery and Aortic Sizes, n = 58

	Pre-HF MRI		Pre-TCPC MRI		
	Mean (SD)	Range	Mean (SD)	Range	p Value
⁎Distal right pulmonary artery, mm^2^/m^2^†	166.2 (76.4)	42.7–371.2	158.8 (56.7)	69.9–299.5	0.006
⁎Proximal left pulmonary artery, mm^2^/m^2^	91.6 (86.2)	15.4–582.9	101.4 (53.8)	26.3–283.5	0.010
⁎Narrowest left pulmonary artery, mm^2^/m^2^	72.4 (54.5)	8.7–282.9	52.1 (25.6)	8.2–128.1	0.001
⁎Narrowest left pulmonary artery:proximal left pulmonary artery, %	81.2 (24.2)	16.9–133.3	55.8 (21.8)	16.6–111.5	0.000
Native aorta, mm^2^/m^2^	63.0 (80.4)	11.1–450.1	55.7 (61.5)	11.2–341.0	0.07
Neo-aorta sinus, mm^2^/m^2^	704.9 (170.7)	400.1–1161.7	680.3 (184.2)	375.9–1505.3	0.10
Ascending aorta, mm^2^/m^2^	533.2 (155.0)	258.2–1001.1	516.4 (145.9)	283.1–915.8	0.22
Transverse arch, mm^2^/m^2^	425.3 (149.6)	189.3–877.9	440.9 (168.4)	226.2–1252.3	0.92
Upper descending aorta, mm^2^/m^2^	163.6 (65.3)	46.4–335.7	177.3 (54.2)	89.6–318.1	0.11
⁎Lower descending aorta, mm^2^/m^2^	148.2 (45.5)	58.9–318.2	112.0 (31.4)	71.3–212.9	<0.001
⁎Upper:lower descending aorta	1.14 (0.38)	0.25–2.24	1.63 (0.48)	0.83–3.50	<0.001

Abbreviations as in Table 2.

⁎ p < 0.05 on Wilcoxon.

† There are limited numbers of measurements of the distal right pulmonary artery after HF (n=28) due to the proximity of the HF anastomosis to the right pulmonary artery branches.

## Appendix

Online Table 1 Pulmonary Artery and Aortic Sizes, n = 58
