# Supplementary material for: Serial Magnetic Resonance Imaging in Hypoplastic Left Heart Syndrome Gives Valuable Insight Into Ventricular and Vascular Adaptation
Source: J Am Coll Cardiol. 2013 Feb 5;61(5):561–70. doi: 10.1016/j.jacc.2012.11.016 (PMC3573231; doi:10.1016/j.jacc.2012.11.016)
Supplement: Online Table 1 — Pulmonary Artery and Aortic Sizes, n = 58 [file mmc1.docx]

**APPENDIX**

**Supplementary Table 1. Pulmonary Artery and Aortic Sizes, n = 58**

|  | **Mean (SD) change in size (unindexed)** | **Percentage with an absolute increase in size** |
| --- | --- | --- |
|  |  |  |
| Distal right pulmonary artery, mm^2^ *(n = 28)*  Proximal left pulmonary artery, mm^2^  Narrowest left pulmonary artery, mm^2^  Native aorta, mm^2^  Neo-aorta sinus, mm^2^  Ascending aorta, mm^2^  Transverse arch, mm^2^  Upper descending aorta, mm^2^  Lower descending aorta, mm^2^ | 22.9 (34.2)  27.6 (37.4)  5.7 (14.3)  11.5 (15.9)  161.7 (109.2)  123.2 (72.3)  116.9 (79.9)  48.1 (31.2)  15.9 (21.9) | 75.0%  89.5%  61.4%  71.9%  98.3%  96.5%  100%  98.3%  77.6% |
| There are limited numbers of measurements of the distal right pulmonary artery after HF due to the proximity of the HF anastomosis to the right pulmonary artery branches making measurement not possible. | | |
